# The Protective Effect of Shen Qi Wan on Adenine-Induced Podocyte Injury

**DOI:** 10.1155/2020/5803192

**Published:** 2020-11-20

**Authors:** Yiyou Lin, Jieying Zhang, Yunbo Fu, Liting Ji, Luning Lin, Hongshu Chen, Yuanxiao Yang, Changyu Li

**Affiliations:** ^1^College of Pharmacy, Zhejiang Chinese Medical University, Hangzhou, Zhejiang 310053, China; ^2^The First Affiliated Hospital of Zhejiang Chinese Medical University, Hangzhou, Zhejiang 310006, China; ^3^School of Basic Medical Sciences and Forensic Medicine, Hangzhou Medical College, Hangzhou, Zhejiang 310053, China

## Abstract

Podocytes are a special type of differentiated epithelial cells that maintain the glomerular filtration barrier in the kidney. Injury or damages in podocytes can cause kidney-related disorders, like CKD. The injury or dysfunction of podocytes can occur by different metabolic disorders. Due to the severity and complexity of podocyte injuries, this state is considered as a serious health issue worldwide. Here, we examined and addressed the efficacy of an alternative Chinese medicine, Shen Qi Wan (SQW), on podocyte-related kidney injury. We evaluated the role and mechanism of action of SQW in podocyte injury. We observed that SQW significantly reduced 24-hour urinary protein and blood urea nitrogen levels and alleviated the pathological damage caused by adenine. Moreover, SQW significantly decreased the expression of nephrin and increased the expression of WT1 and AQP1 in the kidney of mice treated with adenine. We observed that SQW did not effectively reduce the high level of proteinuria in AQP1^−/−^ mice indicating the prominent role of AQP1 in the SQW-ameliorating pathway. Transmission electron microscopy (TEM) images indicated the food processes effacement in AQP1^−/−^ mice were not lessened by SQW. In conclusion, podocyte injury could alter the pathological nature of the kidney, and SQW administration relieves the nature of pathogenesis by activating AQP1.

## 1. Introduction

Chronic kidney disease (CKD), designating the gradual loss of kidney function, is common in people aged beyond 65 years. CKD is considered being a serious health issue worldwide [1]. The global escalation of CKD is mainly driven by disorders such as diabetes mellitus, hypertension, obesity, and aging. The severity of CKD could lead towards the syndromes like proteinuria.

As a prominent marker for CKD, proteinuria could describe the severity of kidney damage or CKD [2–4].

The anatomy of the kidney consists of a specialized differentiated epithelial cell-podocyte. The podocytes are located in the lateral glomerular basement membrane and endotheliocytes and make the lining of the glomerular filtration barrier [5]. In CKD or kidney disease, podocyte dysfunction can relate to proteinuria and glomerular diseases [2, 6–8]. In podocytes, nephrin acts as a major component of the slit diaphragm [9]. Downregulation of nephrin expression in podocytes induces cellular damage and that contributes to proteinuria [10]. To maintain the differentiated state of podocytes, WT1, a transcription factor, takes part in a major role [11]. Studies showed that the silencing of WT1 in podocytes of adult mice results in proteinuria and glomerulosclerosis [12]. WT1 binds to different target genes to activate the regulatory pathway in kidney development. WT1 loss-of-function approach in kidney explants arrest the development of nephron progenitors, thereby arresting the kidney development [13]. The importance of nephrin and WT1 in the context of CKD needs to be examined. The crucial markers such as nephrin and WT1 with a specific treatment protocol for CKD were examined in this article.

Shen Qi Wan (SQW) is a classic recipe in the clinical treatment of chronic kidney disease, which was recorded in Zhang Zhongjin's Jingui Yaolue [14]. The formula consists of Dihuang (Radix Rehmanniae), Danpi (Cortex Moutan), Zhuyu (Dogwood), Fulin (Poria cocos), Shanyao (Yam), Zhexie (Alisma), and Rougui (Monkshood). SQW is widely used in many kinds of diseases and achieve its intended effects [15–17]. Experimental studies showed treatment with SQW improved the renal injury in various kidney diseases [18, 19]. In our previous study, SQW could effectively reduce the level of urinary protein and relive renal pathological injury induced by adenine in rats [20]. Previous work also showed that SQW can ameliorate the renal fibrosis in model rats caused by adenine [21]. But the effect of SQW in proteinuria though ameliorating podocyte injury is still unclear. Additionally, SQW treatment was able to ameliorate the kidney injury in rats also increased the expression of AQP1. The role of AQP1 (aquaporin 1) in SQW-mediated renal care needs to be evaluated in terms of ameliorating podocyte injury.

AQPs are a group of transmembrane channels that mainly regulate the isotonicity or fluid balance in the body [22]. AQP1 is abundantly expressed in the renal tissue and plays a crucial role in renal function to maintain the fluid balance [23, 24]. The main function of AQP1 is constitutive absorption of water in the glomerular filtrate, and it has a profound role in epithelial cell proliferation, cell migration, and angiogenesis [25, 26]. During proteinuria pathogenesis, the expression of AQP1 was downregulated in various occasions [27–29]. However, to date, little is known regarding the role of AQP1 in the progress of podocyte injury and proteinuria. To test this hypothesis, we investigated the potential effects of SQW on adenine-induced podocyte injury. Moreover, we used AQP1^−/−^ knockout mice to address the role of SQW on podocyte injury via adenine-induced pathogenesis.

## 2. Materials and Methods

### 2.1. Animals and Groups

The animal experiments were performed with humanity and under the approval of the “Ethics of Committee of Zhejiang Traditional Chinese Medical University” (ZSLL-2018-012). The male C57BL/6 mice were housed in an SPF-grade facility (age: 12 weeks; weight: 22 ± 4 g) and were obtained from Shanghai Xipuer-Bikai Experimental Animal Co., Ltd (laboratory rearing room permit no. SCXX (Shanghai) 2018–0006). All mice were housed at a constant room temperature of 20 ± 2°C with 50–60% humidity and supplied with standard diet and water.

The health status of each mouse was confirmed, and minimum weight reached 20 g before the study began. A total of 18 male mice (C57BL/6) were randomly divided into the control group (*n* = 6), adenine-treated group (*n* = 6), and SQW treatment group (*n* = 6). The dosage of adenine was 50 mg/kg BW/day. Except for the control group, the remaining two groups were supplemented with adenine dose (lot: C10129556; Shanghai Macklin Biochemical Co., Ltd.) for two weeks. After two weeks of adenine administration, Ade + SQW group mice were supplemented with SQW (lot: 160402; Zhongjing Wanxi Pharmaceutical Co., Ltd.) at a dose of 3.0 g/kg BW/day for three weeks. The composition of SQW including seven medicinal herbs are listed in Table 1. The control group and adenine group (Ade group) were supplemented with the same volume of water.

AQP1^−/−^ mice and AQP1^+/+^ mice on C57BL/6 background were littermates that were established by CRISPR/Cas9 gene-editing technology [30]. Before the initiation of the study, the genotype of each mouse was confirmed by PCR analysis of DNA, and minimum weight reached 25 g. The male mice at 4 months were grouped into the wildtype (WT) (AQP1^+/+^, *n* = 5), AQP1^−/−^ (AQP1-KO) group (*n* = 8), and SQW-treated (3.0 g/kg BW/day) AQP1^−/−^ (AQP1-KO) group (*n* = 8). The experiments were performed according to the proper guidelines and with adequate humanity. At the experimental endpoint, all mice were anaesthetized by intraperitoneal injection of sodium pentobarbital at a dose of 50 mg/kg. Then, carbon dioxide was used to euthanize the mice. During the whole experimental procedure, we tried our best to reduce the suffering of mice. The kidney samples were excised and kept in liquid nitrogen before tests.

Before sacrifice, the body weight of all the mice was measured. Later, each mouse was placed in an individual metabolic cage with plenty of food and water, and then food and water intake and urination were recorded for 24-hour.

### 2.2. Biochemical Analysis of Blood and Urine

Blood form the mice were obtained from heart puncture after anesthesia. The serum was isolated from the blood by centrifuging at 3000 rpm for 10 min at 4°C. The urine samples were collected from metabolism cages and centrifuged at 3000 rpm for 5 min at 4°C. All the samples were stored at −80°C. The 24-hour urine total protein (U-TP), blood urea nitrogen (BUN), and serum creatinine (Scr) were detected by using an automatic biochemical detector (Hitachi Co., Tokyo, Japan).

### 2.3. Hematoxylin and Eosin (H&E) Staining

The kidney tissues were fixed in 10% buffered formalin and embedded in paraffin. Then, the kidney tissues were sectioned using a microtome (RM2245, Leica Biosystems, USA). The sections were stained with hematoxylin and eosin to evaluate the renal structure. Photomicrographs of H&E sections were randomly taken at 200x magnification under the digital pathology scanner (VS120–S6–W, OLYMPUS, Japan).

### 2.4. Immunofluorescence Staining

Immunofluorescence staining was performed on paraffin sections. The tissue sections were processed for the primary antibody incubation [31]. The primary antibodies against nephrin (1 : 500, Abcam, Cambridge, UK), WT1 (1 : 50, Abcam, Cambridge, UK), and AQP1 (1 : 200, Abcam, Cambridge, UK) were incubated at 37°C for 1 h, followed by the incubation with secondary antibodies (goat anti-rabbit lgG, RS23220, ImmunoWay, USA) for 1 h at 37°C. The sections were counterstained with DAPI (D9542, Sigma, USA) to visualize the nuclei and observed using a digital pathology scanner (VS120–S6–W, OLYMPUS).

### 2.5. Western Blot

Total protein from the kidney tissues was isolated after being lysed by RIPA buffer (01408, Beyotime Biotechnology, China) containing PMSF, protease, and protein phosphatase inhibitor for 30 min on ice. All samples were centrifuged at 12000 g for 15 min (4°C). Protein concentration was determined using the BCA assay kit (P0012, Beyotime Biotechnology, China). An equal amount of protein (40 *μ*g) was subjected to 8–10% SDS-PAGE gel and then transferred to PVDF membranes (162–0177, BIO-RAD, USA). The membranes were blocked with 5% BSA and incubated overnight with the following antibodies at 4°C: antinephrin (ab216341, Abcam, Cambridge, UK), anti-Wilms Tumor 1 (WT1) (ab89901, Abcam, Cambridge, UK), anti-Aquaporin 1 (ab168387, Abcam, Cambridge, UK), and anti-GAPDH (EM1101, Huaan Biotechnology, Hangzhou, China). After incubating with peroxidase-conjugated secondary antibodies (goat anti-rabbit-antibody, LI-COR Biosciences, USA), the protein expression bands were developed with chemiluminescence. The densitometry of the brands was then calculated using the Odyssey near-infrared dual-color laser imaging system (Odyssey Clx, LI-COR Biosciences, USA).

### 2.6. Transmission Electron Microscopy

The renal cortices were collected from each group (1 mm^3^) and fixed in 2.5% glutaraldehyde for 24 h at 4°C. Then, samples were washed with phosphate-buffered saline (PBS) three times and postfixed with 1% osmic acid for 2 h. After dehydrating in gradient acetone and ethanol, each ultrathin section (50–70 nm) was stained with uranyl acetate and lead citrate and examined and photographed at 15000x magnification in an electron microscope (H-7650, HITACHI, Japan).

### 2.7. Statistical Analysis

The results were expressed as mean ± standard deviation (SD). GraphPad Prism version 8 (GraphPad Software, CA, USA) and SPSS software version 22.0 (IBM Corporation, Chicago, IL, USA) was used for the analysis of the significance between different groups by one-these factors with adenine if *P* < 0.05.

## 3. Result

### 3.1. SQW Treatment Reduced the Renal Injury Markers in Mice Caused by Adenine

**Figure 1:**
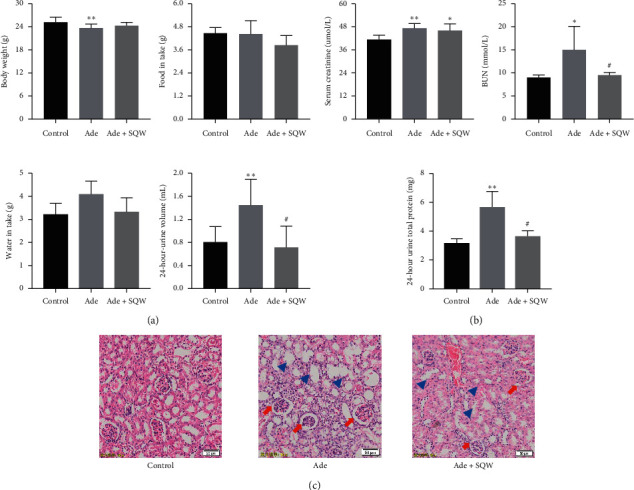
Effect of SQW on kidney injury of C57BL/6 induced by adenine. (a) The effect of SQW on body weight, food intake, water intake, and 24-hour urine volume in C57BL/6 mice (*n* = 5). (b) The effect of SQW on the level of Scr, BUN, and 24-hour UTP. (c) Hematoxylin-eosin (H&E) staining showing the renal structure in C57BL/6 mice after the administration of SQW. Scale bars, 50 *μ*m. Data are expressed as mean ± S.E.M. (*n* = 3). ^*∗*^*P* < 0.05, ^*∗∗*^*P* < 0.01 vs. control group, and ^#^*P* < 0.05 vs. Ade group.

The pathological markers for any pathogenesis reflect the body weight, food and water intake, and 24-hour urine volume. In this study, we examine these factors following adenine introduction. There were significant changes in the water intake and urine production with adenine treatment, but no significant changes were observed for body weight and food intake (Figure 1(a)). In contrast, SQW administration on adenine-treated mice relatively decreased the urine production and water intake (*P* < 0.05). For the food and water intake, there was no significant difference among the control group, the Ade group, and the Ade + SQW group. Later, we examined different pathological markers, to understand the efficacy of SQW on adenine-treated mice. We detected the level of BUN, serum creatinine, and 24-hour urine total protein (U-TP) by an automatic biochemical detector. As shown in Figure 1(b), the level of the Scr, BUN, and 24 h-UTP were significantly elevated in the adenine-treated group while compared with the control group (*P* < 0.01). SQW treatment decreased BUN and 24-hour UTP (*P* < 0.05) level. However, the level of Scr seems to possess no significant difference after SQW treatment. And the changes pathological markers revealed that adenine was toxic for the kidney, and SQW was protecting the kidney from the pathological damages caused by adenine. Next, H&E staining was performed to examine the morphological changes in renal tissue with Ade and Ade + SQW (Figure 1(c)). The distortion in renal morphology was observed in the Ade group, which includes renal tubules ectasia (blue arrowheads), dilated renal capsule, glomerular necrosis, and segmental thickening of glomerular necrosis (red arrows). In contrast with adenine administration, SQW has significantly alleviated the severity of pathological damages to a certain degree. Moreover, the downregulated proteinuria suggests SQW might reduce the podocyte injury to a certain degree.

### 3.2. SQW Treatment Reduced the Podocyte Injury Caused by Adenine

**Figure 2:**
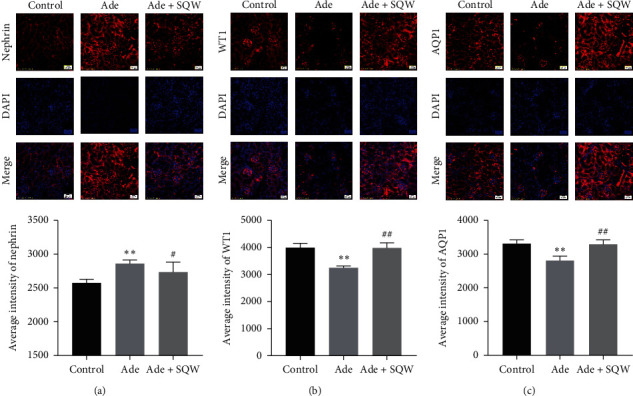
Immunofluorescence staining of podocyte-associated molecules and AQP1 in the renal cortex of the C57BL/6 mice. Immunofluorescence images of (a) nephrin staining and intensity analysis at the glomerulus of the C57BL/6 mice, (b) WT1 staining and intensity analysis at the glomerulus of the C57BL/6 mice, and (c) AQP1 staining and intensity analysis at glomerulus of the C57BL/6 mice. Scale bars, 20 *μ*m. Data are expressed as mean ± S.E.M. (*n* = 3). ^*∗*^*P* < 0.05, ^*∗∗*^*P* < 0.01 vs. control group, ^#^*P* < 0.05, and ^##^*P* < 0.01 vs. Ade group.

**Figure 3:**
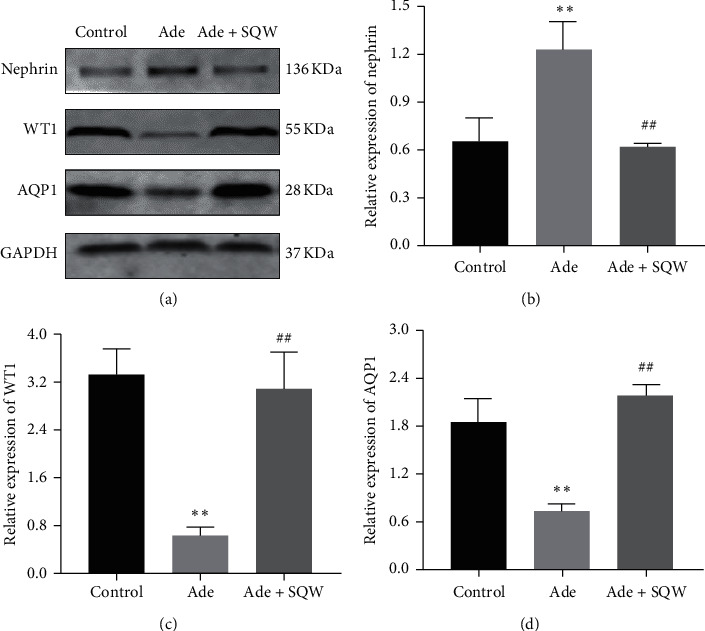
Effect of SQW on the expression of podocyte-associated molecules and AQP1 in the renal cortex of the C57BL/6 mice. (a) Protein bands of nephrin, WT1, and AQP1 by western Blot. (b) Nephrin, WT1, and AQP1 protein expression relative density to glyceraldehyde 3-phosphate dehydrogenase (GAPDH). Data are expressed as mean ± S.E.M. (*n* = 3). ^*∗*^*P* < 0.05, ^*∗∗*^*P* < 0.01 vs. control group, ^#^*P* < 0.05, and ^##^*P* < 0.01 vs. Ade group.

To explore the ameliorating effect of SQW on the podocyte injury caused by adenine, the localization, and expression of podocyte-associated molecules were detected by immunofluorescence staining and Western blot. As seen in Figure 2, the intensity of nephrin, WT1, and AQP1 were analyzed in glomerulus by immunofluorescence. Nephrin expression was elevated with adenine when compared with the control group. On the other hand, SQW treatment significantly reduced the expression of nephrin compared with the Ade group (Figure 2(a)). We examine the expression of WT1 and AQP1 and was found to be decreased in the Ade group from the control group. After the administration of SQW, the expression of WT1 and AQP1 was significantly increased (Figures 2(b) and 2(c)) in comparison with the Ade group. As shown in Figures 3(a) and 3(b), the expression of nephrin, WT1, and AQP1 was examined by western blot. The expression of nephrin was upregulated in the Ade group, but the expression of WT1 and AQP1 was downregulated with Ade. Treatment with SQW elevated the expression of WT1 and AQP1, but nephrin expression was downregulated significantly.

### 3.3. SQW  Treatment on  AQP1^−/−^ Mice

**Figure 4:**
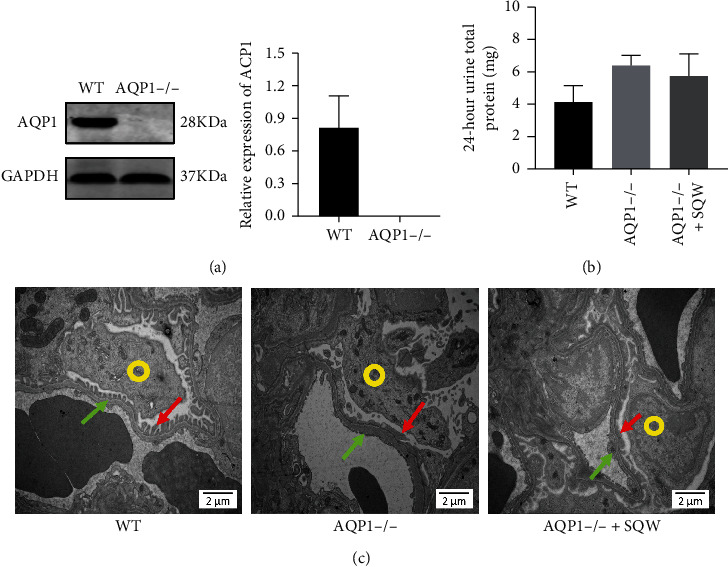
Effect of SQW on proteinuria and ultrastructure of kidney tissue in AQP1^−/−^ mice. (a) The expression of AQP1 in the kidney cortex in AQP1^−/−^ mice. (b) The effect of SQW on 24-hour urine total protein of AQP1^−/−^ mice. (c) The effect of SQW on kidney ultrastructure assessed by transmission electron microscopic analysis in AQP1^−/−^ mice (the yellow circle is the podocyte soma, the red arrow points to the podocyte foot process, and the green arrow points to the renal basement membrane). Original magnification x15 000; scale bars, 2 *μ*m (*n* = 5). Data are expressed as mean ± S.E.M. (*n* = 5). ^*∗*^*P* < 0.05, ^*∗∗*^*P* < 0.01 vs. WT group, and ^#^*P* < 0.05 vs. AQP1^−/−^ group.

To validate the role of AQP1 on SQW treatment of podocyte injury, we established AQP1^−/−^ mice by CRISPR/Cas9 gene-editing technology. AQP1 expression was diminished in AQP1^−/−^ mice in contrast with wildtype (WT) mice (Figure 4(a)). As shown in Figure 4(b), the higher level of 24-hour urine total protein was observed in AQP1^−/−^ mice than WT mice. Moreover, SQW was not elevated in 24-hour urine total protein level in AQP1^−/−^ mice, which suggested that AQP1 was involved in the SQW treatment of podocyte injury.

To assess ultrastructural changes in the podocyte morphology of AQP1^−/−^ mice, electron microscopy was performed (Figure 4(c)). The glomerular ultrastructure picture shows that the glomerular structure of WT mice is clear and complete, and the foot processes are neat and visible (as the red arrow indicates). But in AQP1^−/−^ mice, the glomerular texture was relatively loose, and fragment-like tissues were diffused at the edges of the main processes of the podocytes (as green arrow points). The fusion of the podocyte processes has occurred, and the structure was blurred. However, the damage of glomerular podocytes in AQP1^−/−^ mice seems not to be reduced at all after SQW seems no different after SQW intervention. Taken together, AQP1 might act as a protective target for alleviating podocyte injury by SQW.

## 4. Discussion

The kidney functions silently diminished during CKD disorder. The CKD disorder is generally prone to old-aged people. CKD onset is due to different disorders such as diabetes, high blood pressure, glomerulonephritis, interstitial nephritis, and polycystic kidney disease. Worldwide, CDK-related death increased enormously in the past decade. In the kidney, the renal cell, namely, podocytes possess an important role in water regulation during kidney filtration. The podocyte injury/damage during CKD onset is common. The damages in podocytes diminish the kidney function. Therefore, the improvement in disease management could be achieved by understanding the relation between podocytes and CKD. The adopted therapy in this study was able to ameliorate the podocyte damage caused by adenine. We described here, the effect of one conventional traditional Chinese medicine, SQW, on an animal model of CKD-related podocyte damage. The beneficial effect of SQW is clearly described in this article, and we delineated the working principle of SQW on podocyte injury in animal models.

Injury to podocytes causes detachment of the cells from the glomerular base membrane. This special state is responsible to cause proteinuria and glomerulosclerosis [32]. Nephrin is specifically expressed on the podocyte fissure membrane of the kidney. Nephrin maintains the integrity of the podocyte fissure membrane [33]. Another transcription factor, WT1, maintains the differentiated state of podocyte and maintains its integrity [34–37]. Evidence indicated that WT1 drives the expression of a series of podocyte-specific genes, such as nephrin, podocyte, and podocalyxin [38, 39]. Hence, by maintaining the basal expression of nephrin and WT1, podocytes could be secured from the damage. Our evidence concluded that SQW improved the podocyte injury state by restoring the abnormal expression of the podocyte-associated protein.

In our study, we investigated that SQW treatment effectively alleviated podocyte injury caused by adenine administration in mice. Adenine is a common agent for inducing chronic kidney injury or CKD [40]. Previously, adenine-fed mouse was shown to possess podocyte injury in glomeruli [41]. Similarly, our observation on adenine-treated mice was affected by excessive proteinuria, histopathological damage in the kidney. We observed the upregulation of the markers like nephrin and downregulation of WT1 in the adenine-treated group. Moreover, massive proteinuria and abnormal expression of podocyte-associated molecules indicated adenine could lead to podocyte injury. SQW, a classic Chinese medicine, is widely used for chronic kidney damage in China [42]. Previous studies suggested that SQW can effectively reduce the level of proteinuria in adenine-induced rats [20]. However, whether SQW on the treatment of proteinuria through relieving podocyte injury is unknown. In this study, we showed that SQW treatment declined the proteinuria state and significantly ameliorated the abnormal expression of nephrin and WT1 in the kidney tissue of adenine-induced mice.

AQP1 is abundantly expressed on the kidney tissue. AQP1 is often dysregulated in many renal disorders and plays an important role in maintaining osmotic balance and fluid metabolism in the kidney [43, 44]. During kidney disease, AQP1 expression used to get downregulated [45, 46]. The structure of the podocytes also gets regulated by AQP1 protein. As in AQP1 KO mice, the ultrastructures of podocytes were altered. In adenine-supplemented mice, SQW treatment elevated the expression of AQP1, and thus AQP1 may play an important role in maintaining the integrity of the podocytes, apart from maintaining the fluid and osmotic balance in the kidney cells. From our observations, we speculated that AQP1 may be a pivotal target for SQW treatment. In AQP1^−/−^ mice, the level of 24-hour proteinuria and ultrastructural changes in podocyte morphology have been observed. The severity of the damages in podocytes was prominently higher in AQP1^−/−^ mice by adenine treatment when compared with wildtype mice. Moreover, SQW was not able to ameliorate the podocyte injury caused by adenine in AQP1^−/−^ mice. These results indicated that AQP1 was a key modulator for SQW's mechanism of action on podocyte injury. This study needs to define further the mechanism behind the activation of AQP1 by SQW in injured podocyte cells.

Taken together, the present study demonstrated the role of SQW in alleviating podocyte injury, by reducing proteinuria and possibly by regulating the expression of AQP1. These findings may explain the importance of Chinese traditional medicine recipes on kidney disease. This study provides an insight into the mechanism of action of SQW on ameliorating podocyte injury. AQP1 may play an important role for this therapeutic purpose.

## 5. Conclusion

In conclusion, SQW could effectively alleviate podocyte injury induced by adenine. In light of podocyte-associated protein expression and kidney injury index analysis, we confirm the effect of SQW in alleviating podocyte injury. Meanwhile, the decreased level of 24-hour UTP and ultrastructural changes of podocytes by TEM was observed in AQP1^−/−^ mice. And there is no difference change after administration of SQW. Collectively, our data demonstrated that SQW could significantly attenuate the podocyte injury possibly via increasing the expression of AQP1.

## Figures and Tables

**Table 1:** The composition of Shen Qi Wan (SQW).

Chinese name	Subfamily	Scientific name	Quantity percentiles (%)
Fuzi	Ranunculaceae	*Aconitum carmichaelii* Debx.	3.70
Rougui	Lauraceae	*Cinnamomum cassia* Presl	3.70
Shudihuang	Scrophulariaceae	*Rehmannia glutinosa* Libosch.	29.63
Shanzhuyu	Cornaceae	*Cornus officinalis* Sieb. et Zucc.	14.82
Shanyao	Dioscoreaceae	*Dioscorea opposita* Thunb.	14.82
Fuling	Polyporaceae	*Poria cocos* (Schw.) Wolf	11.11
Mudanpi	Ranunculaceae	*Paeonia suffruticosa* Andr.	11.11
Zexie	Alismataceae	Alisma orientalis (Sam.) Juzep.	11.11

## Data Availability

The datasets used and analyzed during the current study are available from the corresponding author on reasonable request.
